# Obstetric violence from the perspective of healthcare personnel: a qualitative systematic review

**DOI:** 10.3389/fgwh.2026.1773729

**Published:** 2026-03-12

**Authors:** Fátima María Guzmán-Guevara, Natalia I. Manjarres-Posada, Georgina Vega-Fregoso

**Affiliations:** 1 Master in Socio-Medical Sciences, Department of Social Sciences, Centro Universitario de Ciencias de la Salud, Universidad de Guadalajara, Guadalajara, México; 2 Regional Institute for Public Health Research (IRISP), Department of Public Health, Centro Universitario de Ciencias de la Salud, Universidad de Guadalajara, Guadalajara, México; 3 Department of Public Health, Centro Universitario de Ciencias de la Salud, Universidad de Guadalajara, Guadalajara, México

**Keywords:** health personnel, midwifery, obstetric violence, obstetricians, perception, qualitative methods, systematic review

## Abstract

**Background:**

While empirical research on obstetric violence has focused primarily on women's experiences, the perspectives of healthcare personnel remain relatively neglected. Consequently, this study synthesized qualitative evidence regarding healthcare professionals’ perceptions of obstetric violence to understand how their views are constructed and which contextual factors influence the reproduction of these practices.

**Methodology:**

A qualitative systematic review was conducted, covering literature published between 2019 and 2025. The search encompassed the ProQuest Central, Sage Journals, Web of Science, Scopus, SciELO, and PubMed databases. Following PRISMA guidelines, 22 studies that met the inclusion criteria and addressed the research question were included. The data were analyzed using a hermeneutic approach oriented toward recovering the meanings attributed to obstetric violence within its specific production contexts.

**Results:**

The reviewed studies reveal tension between recognizing and denying obstetric violence among professionals. Some discourses justify institutionalized practices under biomedical logic, while others express discomfort and internal contradictions toward actions perceived as violent yet considered part of the clinical routine. Medical hierarchy, professional training, work overload, and lack of awareness of reproductive rights were identified as factors shaping these perceptions. Differences were also observed between disciplines (e.g., midwifery vs. obstetrics) and levels of experience (e.g., professionals vs. students).

**Discussion:**

Findings suggest that obstetric mistreatment is a systemic and modifiable phenomenon rather than merely a product of individual intent. Empirical intervention literature demonstrates that professional perceptions can be shifted through structured institutional reforms and training. Eradicating obstetric violence requires moving towards a systemic thinking approach that addresses institutional stressors and promotes humanized, woman-centered care models.

## 1 Introduction

Obstetric violence is defined as any action or omission that causes physical, psychological, or emotional harm to women during pregnancy, childbirth, or the postpartum period. It represents a violation of fundamental human rights and has serious implications for maternal and neonatal health (1). According to the 2021 National Survey on the Dynamics of Household Relationships (ENDIREH), 31.4% of Mexican women aged 15 to 49 who gave birth or underwent a C-section in the last 5 years experienced abuse by their caregivers (2). This figure may be higher when considering the normalization of symbolic violence in delivery rooms (3).

Research of the mistreatment of women during childbirth worldwide dates back to the 1980s and was primarily conducted by Anglo-Saxon researchers, who referred to it as “disrespect and abuse”. The term “obstetric violence,” however, was coined in Latin America and officially used for the first time in Venezuela's Law on Women's Access to a Life Free of Violence, which took effect in March 2007. Mexico incorporated the term into the Law on Women's Access to a Life Free of Violence for the state of Veracruz in 2008 (4). Globally, the World Health Organization (WHO) recognized obstetric violence in 2014, followed by the United Nations (UN) in 2019 (5).

Interest in this phenomenon has grown, and it has been analyzed from various perspectives. An extensive bod**y** of research demonstrates the severe physical and emotional impacts of obstetric violence on pregnant individuals (6, 7). Notable contributions include legal approaches, classification typologies, the validation of perception scales, and numerous studies within the social sciences and humanities. Another line of research explores the perspective of health professionals, which is crucial for a holistic understanding of all stakeholders in this complex scenario. However, this area remains significantly less developed than studies focused on users' experiences, and few proposals seek to address and transform this problem (8).

Therefore, it is essential to deepen the analysis of obstetric violence from the perspective of healthcare personnel. This will facilitate the identification of its manifestations, understand the interpretive frameworks that underpin it, recognize the ethical tensions faced by professionals, and acknowledge the institutional factors that perpetuate it. The limited attention given to this viewpoint has hindered the development of integrated strategies to transform the obstetric care model. Thus, this systematic review aims to analyze the findings of recent qualitative studies addressing healthcare personnel's perceptions of obstetric violence to understand how these views are formed and which contextual factors influence its reproduction.

## 2 Methodology

This qualitative systematic review of literature published between 2019 and 2025 aims to understand obstetric violence from the perspective of healthcare personnel. Data were analyzed using thematic synthesis, following the framework proposed by Thomas and Harden (9), which facilitates the integration of qualitative findings through the identification and organization of emerging themes. This methodology was selected for its capacity to generate conceptual categories from primary data while preserving original interpretive frameworks. Furthermore, it enables an analytical reconstruction that accounts for cross-cutting patterns and discursive tensions among healthcare providers.

### 2.1 Search strategy

The exhaustive, pre-planned search strategy was implemented to identify all relevant studies (10). Six electronic databases were consulted: ProQuest Central, Sage Journals, Web of Science, Scopus, SciELO, and PubMed. The search encompassed articles published between January 2019 and June 2025, utilizing the following descriptors and Boolean operators: (“obstetric violence” OR “mistreatment during childbirth”) AND (“health personnel” OR “healthcare professionals”) AND (“qualitative research” OR “interviews” OR “focus groups”) AND (“perceptions” OR “views” OR “experiences”) AND (“healthcare systems”). Inclusion filters were applied for language (English, Spanish, and Portuguese) and publication timeframe.

### 2.2 Inclusion and exclusion criteria

Studies employing qualitative or mixed-methods designs with qualitative analyses that explicitly addressed healthcare personnel's perceptions of obstetric violence were included. Only full-text articles available in English, Spanish, or Portuguese were considered. Exclusion criteria applied to purely quantitative studies, previous narrative or systematic reviews, gray literature, postgraduate theses, and book chapters.

### 2.3 Selection and evaluation of studies

**Figure 1:**
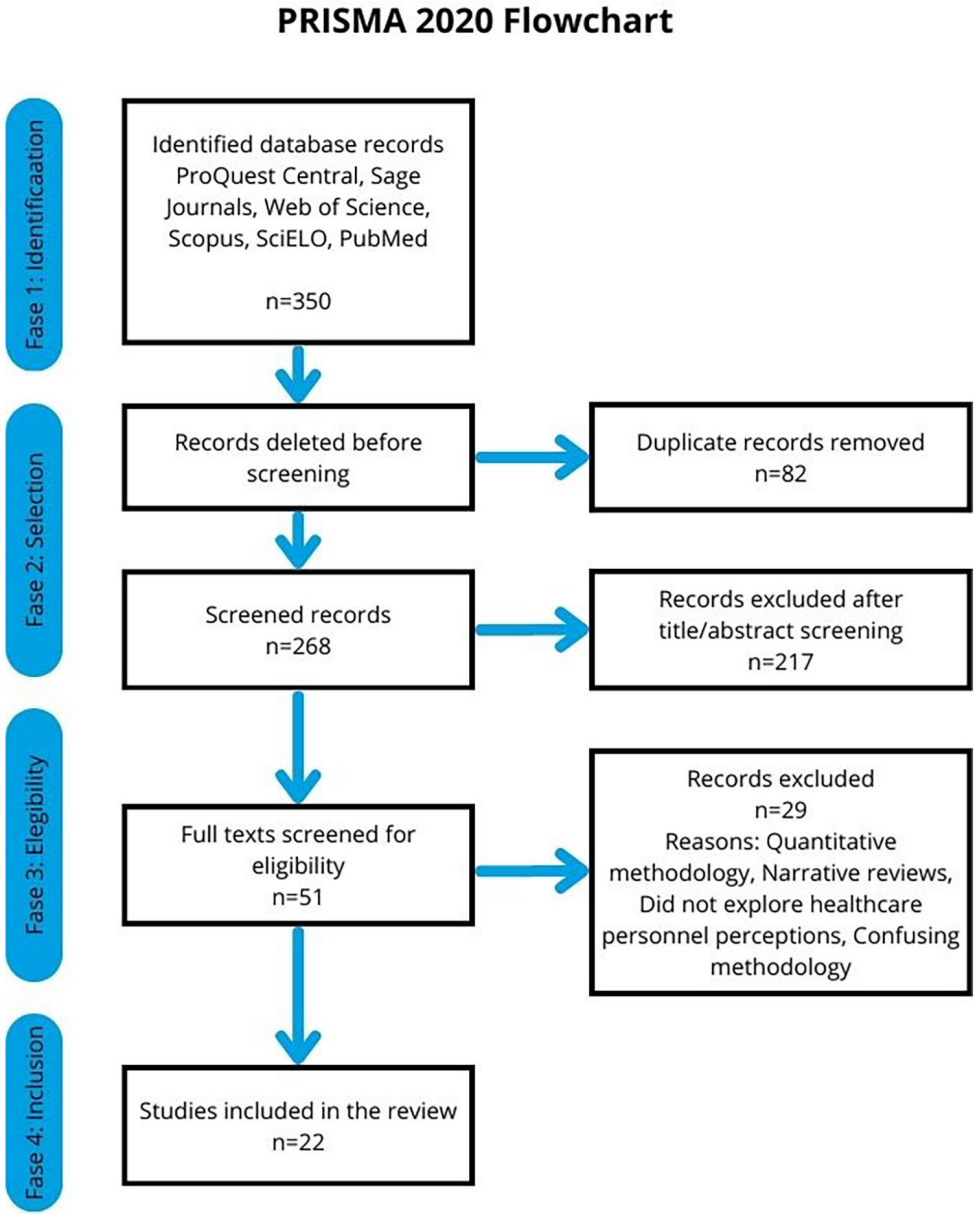
PRISMA 2020 flow diagram illustrating the identification, screening, eligibility assessment, and inclusion of studies in the review.

The initial search yielded 350 records. After removing 82 duplicates, 268 titles and abstracts were screened, from which 51 articles were selected for full-text assessment. Ultimately, 22 studies that met the established criteria and directly addressed the research question were included. Two researchers independently performed the selection process, resolving discrepancies through discussion and consensus. This process is illustrated in the PRISMA 2020 Flow Diagram (Figure 1).

### 2.4 Quality assessment

Methodological quality was evaluated using the Critical Appraisal Skills Programme (CASP) checklist for qualitative research. This tool comprises ten items that assess the validity of results, methodological transparency, and the relevance of findings. Two reviewers independently performed the appraisal, establishing a threshold of 8/10 to define high-quality studies. Discrepancies were resolved through consensus. While no studies were excluded based on this assessment, the level of methodological rigor was documented as contextual information to inform the interpretation of results.

### 2.5 Data extraction and analysis

Data were extracted from the Results and Discussion sections of the selected studies. MAXQDA qualitative software and Excel spreadsheets were utilized to manage and organize the information. Two researchers collaboratively performed line-by-line inductive coding, subsequently grouping these codes into themes and subthemes that reflected the meanings attributed to obstetric violence by healthcare personnel.

The comparative analysis was conducted both within and across studies to identify convergences, tensions, and variations based on participants' professional profiles and training levels. This thematic derivation aimed to capture common discursive patterns and significant disciplinary differences (e.g., obstetric nursing, midwifery, and gynecology-obstetrics) and experience levels (professionals vs. students), while maintaining the interpretive transparency essential to qualitative syntheses.

### 2.6 Ethical considerations

This research did not require evaluation or approval by an ethics committee, as it is a systematic review of existing literature. Consequently, the study did not involve fieldwork, direct interaction with human participants, or the use of personal or sensitive data. The analysis was based exclusively on data from peer-reviewed studies available in academic databases, all of which had undergone their respective ethical review processes during their original implementation.

## 3 Results

This systematic review included 22 qualitative studies published between 2019 and 2025, exploring obstetric violence from the perspective of healthcare personnel across diverse sociocultural and clinical contexts. The included literature varied in participant profiles, encompassing obstetrics and gynecology specialists, obstetric nurses, midwives, and trainees, as well as in geographical regions and specific qualitative methodologies. This diversity provides a comprehensive and contextualized understanding of the phenomenon.

Table 1 summarizes the core characteristics of the included studies, including country of origin, publication year, study population, data collection techniques, and key findings. The predominance of Latin American research situates these findings within a specific regional discourse. Most studies were conducted with practicing health professionals and primarily employed qualitative designs, facilitating an identification of the origins of these accounts and inviting reflection on the conditions under which obstetric violence is reproduced. Furthermore, Table 2 presents the methodological quality assessment of the 22 studies conducted via the CASP tool.

**Table 1:** Summary of the characteristics of the studies included in the systematic review.

Reference	Country/location	Aim of the study	Participants	Method/Qualitative design	Data collection technique	Analysis technique	Key findings
Alejandría et al. (12)	Brazil	Assess the knowledge of nursing professionals involved in childbirth care about obstetric violence.	10 nurses	Qualitative	Semi-structured interview	Bardin content analysis	1) Professionals’ knowledge of the concept of obstetric violence. 2) Practices, techniques, and maneuvers that they consider to be obstetric violence. 3) Rights of women in labor.
Bitencourt et al. (22)	Itajubá, Brazil	Understanding the perception of obstetric violence among professionals who assist in childbirth and delivery	22 nurses, midwives, nursing technicians, and obstetricians, in the private or public sector.	Qualitative	Semi-structured interview	Bardin content analysis	1) Process of change. 2) Respect for physiology and intervention when necessary. 3) Aggressive effect of words and result of interaction between the woman in labor and the team. 4) Lack of preparation of professionals and institutional problems. 5) Failure to recognize damage.
Damas et al. (14)	Artemisa, Cuba	Identify manifestations of obstetric violence that occur during childbirth and the perceptions that women and health professionals have of the phenomenon.	12 women who had had a physiological birth, 10 obstetric nursing professionals, and 10 obstetricians	Qualitative	Semi-structured interviews and non-participant observation	Grounded Theory	1) Perception of VO: one group felt insulted and identified certain behaviors and attitudes as characteristic of the specialty, while another group acknowledged their existence and feared that they were engaging in them. 2) Interventions: the risks to women of not performing them and the pressure of possible complications. 3) Attitudes of health professionals. 4) Regulations established by the health system.
Flores-Romero et al. (16)	Baja California, Mexico	Analyze nursing staff perceptions of mistreatment of women during pregnancy and childbirth.	23 nursing staff members	Qualitative	Interviews	Discourse analysis	1) Direct violence (physical, sexual, psychological, or verbal). 2. Indirect violence (medicalization, neglect, classifying women as fearful, demanding, underage, difficult, without prenatal care, and institutional factors).
Gomes et al. (11)	Brazil	Understanding the structure of nursing students’ social representations of obstetric violence	117 nursing students	Qualitative	Free evocation of words	Creating a chart of the four houses with Prototypical Analysis	1) Core Elements of Social Representation: a) Disrespect linked to the attitudinal dimension of representation, reflecting a negative judgment of professionals’ actions. b) Suffering is related to the affective dimension and reflects the emotional impact and consequences that obstetric violence has on women. c) Violation is understood as the behavior, practice, and actions of professionals that violate the rights of women and their bodies. 2) Perception of Professional Practices: negligence, lack of humanization of care, imposition of procedures, and abuse of power.
Haseli et al. (21)	Iran	Exploring midwifery students’ experiences regarding threats to women's dignity during childbirth	32 midwifery students	Qualitative	In-depth interviews	Conventional content analysis	1) Professional incompetence (Interventions without scientific basis, normalization of disrespect and abuse, justification of violence). 2) Abuse of power (Authoritarian tone, humiliation, hitting the mother, discrimination). 3) Caring only for physical health and not mental health. 4) Structural problems within the system (high workload, staff fatigue, inappropriate environment, disregard for dignity through policies, processes, and laws, facilitation of disrespect and abuse in teaching hospitals).
Martín-Badia et al. (29)	Catalonia, Spain	Delving into midwives’ experiences to describe the ethical perspectives of obstetric violence	24 midwives	Qualitative	Focus groups	Phenomenological analysis	1) The harmfulness of forgetting women's vulnerability. 2) Beneficence requires respect for women's integrity and dignity. 3) They are taking away our autonomy. 4) A problem of social justice towards us, women.
Martín-Castañeda (24)	Barcelona, Spain	Understanding the perceptions of midwives in Barcelona regarding obstetric violence	8 midwives	Qualitative ethnographic	Semi-structured interview	Discourse analysis	1) The existence of obstetric violence. 2) Criticism of the concept of “obstetric violence.” 3) Related factors: the risk-based approach of biomedicine, the pathologization of reproductive processes, and hospital hierarchies. 4) Practices associated with obstetric violence: The Kristeller maneuver as a point of conceptual debate.
Mattei et al. (32)	Brazil	Understanding the perceptions of women and healthcare professionals regarding childbirth care in a university hospital.	38 participants. At least 30 professionals are estimated, including nurses, nursing technicians, residents, and obstetricians on staff.	Qualitative	Semi-structured interview	Grounded Theory	1) Limited physical structure for adequate care. 2) Observing women's (lack of) knowledge about their birthing process. 3) Guidance and care during the birthing process. 4) Guidance on professional training. 5) Discussing new practices and resistance to change with staff physicians. 6) Revealing conflictive obstetric care: good practices and obstetric violence.
Mayisela et al. (13)	South Africa	Explore midwives’ perspectives on the undignified care of women.	7 midwives	Qualitative ethnographic	In-depth interviews and non-participant observation	Inductive analysis	1) Women's lack of bodily autonomy. 2) Structural challenges in the provision of quality maternity care. 3) Obstetric violence: physical, verbal, and emotional abuse, refusal to provide pain medication.
Mayra et al. (20)	India, Switzerland, and United Kingdom	Investigate the underlying causes of mistreatment of women during childbirth based on the perceptions and experiences of midwifery and nursing leaders.	34 midwives and nurse leaders	Qualitative	In-depth interviews	Thematic analysis	1) Reactions to the illustrated painting: Difference in perception between participants from India and elsewhere regarding the severity of abuse, possibly due to different conditioning and exposure to the culture of violence and expectations of care. 2) Factors behind disrespect and abuse during childbirth: Related to the woman (individual and social). Related to the birth attendant (individual, birth environment, political environment). 3) Intersectionality of factors
Menezes et al. (26)	Brazil	Understanding the perception of residents in Obstetric Nursing regarding obstetric violence	15 obstetric nursing residents	Qualitative	Focus groups	Bardin content analysis	1) Inappropriate childbirth care practices. 2) Unnecessary procedures for educational or iatrogenic purposes. 3) Gender, race/ethnicity, and socioeconomic class biases.
Poo et al. (19)	Temuco, Chile	Characterize midwives’ representations of obstetric violence	9 midwives	Qualitative	Interviews	Descriptive analysis	1) Characteristics of VO (definition and manifestations). 2) Factors facilitating VO (archaic training, user characteristics, institutional factors). 3) Preventive factors (training, sanctions, empathy, self-criticism).
Progianti et al. (18)	Rio de Janeiro, Brazil	Identify nursing students’ experiences of suffering and pleasure in relation to work organization.	13 midwifery students	Qualitative	Semi-structured interview	Content analysis	1) Lack of recognition for the work of obstetric nurses. 2) Identification of situations of obstetric violence: Discrepancy between “prescribed work” and “actual work.” 3) Recognition of the activities carried out by students during their internship by women and their families.
Reyes-Amargant et al. (27)	Cataluña España	Explore the factors that influence respectful maternal care and the phenomenon of obstetric violence, as perceived by mothers, midwives, and obstetricians.	8 mothers, 4 midwives, 4 obstetricians	Qualitative	In-depth interviews	Phenomenological	1) Offensive term and definition. 2) VO may be the result of a lack of knowledge among healthcare professionals or the low level of evidence that is sometimes applied in obstetrics. Its existence and use are recognized. 3) Silent VO. 4) Difficulty in multidisciplinary work.
Rodrigues (25)	Brazil	Analyze the values expressed by healthcare professionals’ perceptions of obstetric violence.	24 obstetricians and 24 obstetric nurses	Qualitative	Phenomenological interviews	Phenomenological based on Max Scheler's Theory of Values	1) Lack of awareness and recognition of obstetric violence. 2) Lack of a conscious process for acquiring new knowledge runs counter to scientific progress and quality of care. 3) Vital, ethical, and scientific values are identified as the basis for safe and qualified practice, acting as protective values against obstetric violence.
Sens and Stamm (28)	Brazil	Identify the perception of obstetricians by delving into aspects of obstetric violence perceived in the dimension of doctor-patient and human relationships.	16 obstetricians, 7 obstetrics residents	Mixed	Questionnaire with open-ended questions and Likert scale	Content analysis	1) Women and doctors: a special human relationship. 2) Doctors as victims of violence: 2.1 Victims of institutional structures; 2.2 Victims of victims. 3) Who chooses? On autonomy, clarification, and decision-making. 4) Suggestions for change: how to prevent obstetric or institutional violence.
Sens and Stemm (30)	Brazil	Assess physicians’ perceptions of obstetric/institutional violence	16 obstetricians, 7 obstetrics residents	Mixed	Questionnaire with open-ended questions and Likert scale	Content analysis	Multidimensional categorization of VO/VI. 1) Individual dimension: Refers to individual professional actions, practices, and behaviors (performing procedures not based on evidence). 2) Institutional dimension: Relates to working conditions and infrastructure (lack of beds). 3) Human relations dimension: Addresses aspects of the interaction between the professional and the patient, manifested in the asymmetry of the human-doctor relationship, especially when there are disagreements in the decision-making process.
Silva et al. (31)	Teresina, Brazil	Constructing the Collective Discourse of Postgraduate Nurses in Obstetric Nursing on Obstetric Violencea	20 postgraduate students of Obstetric Nursing	Qualitative and descriptive	Semi-structured interview	Discourse of the Collective Subject	1) The importance of obstetric violence in the academic training of nurses. 2) The profile of the obstetric nurse qualified in humanized care. 3) The applicability of scientific knowledge as a strategy in women's health care. 4) Obstetric violence from the perspective of the health team, reducing unnecessary interventions and implementing good practices, overcoming the view of childbirth as an illness.
Snyder (23)	New Brunswick, USA	Understanding how doulas and midwives experience witnessing obstetric violence	17 doulas and midwives	Qualitative	Semi-structured interview	Thematic analysis	1) Being a Witness: Definition, manifestation, and spectrum of VO. 2) The dilemma of intervention: The social location, positionality, and marginalized identity of doulas and midwives. 3) Emotional and professional impact: Labor is rewarding but incredibly difficult. 4) Fighting the system: mechanisms of “voice and exit.”
Trajano and Barreto (17)	Belém do Pará, Brazil	Analyzing obstetric violence from a gender perspective, based on the accounts of healthcare professionals who assist in childbirth.	20 participants, 6 obstetric nursing residents, 7 gynecology and obstetrics residents, 4 obstetrician-gynecologists, 3 obstetric nurses	Qualitative	Semi-structured interview	Bardin content analysis	1) Physical Abuse: Unnecessary interventions performed without the woman's consent, violating her right to bodily integrity and nullifying her role in the birthing process. 2) Psychological Abuse: Blaming the woman and threatening to abandon her demonstrates a relationship of hierarchy and domination of the professional over the patient. 3) Verbal Abuse: Aggression, statements intended to coerce, ridicule, or belittle women in labor. 4) Restriction of Movement and Position During Childbirth
Yalley (15)	Ghana	Exploring midwives’ perspectives on the drivers of obstetric violence	30 midwives	Qualitative, phenomenological	In-depth interviews	Braun and Clarke's thematic analysis	1) Types of violence observed: physical, verbal, neglect, procedures without consent, discrimination, stigmatization, demanding payments, restricting accompaniment, denying position changes. 2) Factors driving the use of violence: pressure on midwives to ensure successful births and avoid blame for negative outcomes, induction of obedience, poor maternal effort, corrective medicine, individual factors, culture of acceptance of violence. 3) Perspectives on VO: necessary and effective strategy, form of help, different interpretation of abuse.

**Table 2:** Quality appraisal of the included studies using the CASP qualitative checklist.

Reference	1. Does the study have a clear objective?	2. Is qualitative methodology appropriate?	3. Is the research strategy appropriate for addressing the question?	4. Was participant recruitment appropriate?	5. Was the data collected properly?	6. Were the relationships between researchers and participants taken into account?	7. Were ethical issues considered?	8. Was the data analysis rigorous?	9. Are the results clear and well supported by the data?	10. How can the findings be applied to other contexts?	Overall assessment
Alejandría et al. (12)	YES	YES	YES	YES	YES	NO	YES	YES	YES	NO	HIGH
Bitencourt et al. (22)	YES	YES	YES	YES	YES	NO	YES	YES	YES	YES	HIGH
Damas et al. (14)	YES	YES	YES	YES	YES	NO	YES	YES	YES	YES	HIGH
Flores-Romero et al. (16)	YES	YES	YES	PARTIAL	YES	NO	YES	YES	YES	YES	HIGH
Gomes et al. (11)	YES	YES	PARTIAL	YES	YES	PARTIAL	YES	YES	YES	YES	HIGH
Haseli et al. (21)	YES	YES	YES	YES	YES	PARTIAL	YES	YES	YES	PARTIAL	HIGH
Martín-Badia et al. (29)	YES	YES	YES	YES	YES	YES	YES	YES	YES	NO	HIGH
Martín-Castañeda (24)	YES	YES	YES	PARTIAL	YES	NO	YES	YES	PARTIAL	NO	MODERATE
Mattei et al. (32)	YES	YES	YES	YES	YES	PARTIAL	YES	YES	PARTIAL	YES	HIGH
Mayisela et al. (13)	YES	YES	YES	YES	PARTIAL	YES	YES	YES	PARTIAL	YES	HIGH
Mayra et al. (20)	YES	YES	YES	YES	PARTIAL	YES	YES	PARTIAL	YES	YES	HIGH
Menezes et al. (26)	YES	YES	YES	YES	PARTIAL	NO	YES	PARTIAL	PARTIAL	YES	MODERATE
Poo et al. (19)	YES	YES	YES	YES	YES	PARTIAL	PARTIAL	YES	YES	YES	HIGH
Progianti et al. (18)	YES	YES	YES	YES	YES	PARTIAL	YES	PARTIAL	YES	YES	HIGH
Reyes-Amargant et al. (27)	YES	YES	YES	YES	YES	PARTIAL	YES	YES	YES	YES	HIGH
Rodrigues (25)	YES	YES	YES	YES	YES	PARTIAL	YES	PARTIAL	PARTIAL	PARTIAL	MODERATE
Sens and Stamm (28)	YES	YES	YES	PARTIAL	PARTIAL	NO	YES	PARTIAL	YES	YES	MODERATE
Sens and Stemm (30)	YES	YES	YES	PARTIAL	PARTIAL	NO	YES	PARTIAL	YES	YES	MODERATE
Silva et al. (31)	YES	YES	PARTIAL	YES	YES	NO	YES	PARTIAL	NO	YES	MODERATE
Snyder (23)	YES	YES	YES	YES	PARTIAL	YES	YES	YES	YES	YES	HIGH
Trajano and Barreto (17)	YES	YES	YES	YES	PARTIAL	NO	YES	PARTIAL	YES	YES	MODERATE
Yalley (15)	YES	YES	YES	YES	YES	PARTIAL	YES	YES	YES	YES	HIGH

The coding and thematic analysis process identified five primary themes reflecting how healthcare personnel perceive, justify, or problematize obstetric violence; these are summarized in Table 3. Each theme and its associated subthemes are detailed below, accompanied by illustrative quotes from the reviewed studies. This approach facilitates a deeper examination of the meanings attributed by healthcare providers and the interpretive frameworks that underpin both their discourses and clinical practices.

**Table 3:** Categories and subcategories identified through thematic analysis that reflect how health personnel perceive and interpret obstetric violence.

CATEGORIES	SUBCATEGORIES
General perception and recognition of obstetric violence	Recognition of the existence of OB Difficulty in self-recognition and denial Normalization and invisibility Controversial terminology
Perceived manifestations and types of obstetric violence	Physical violence Verbal and psychological violence Negligence and failure to provide care Stigmatization and discrimination Excessive interventionism and medicalization
Factors contributing to its reproduction	Pressure from the healthcare system and poor working conditions Academic training and professional knowledge Attitudes and beliefs of professionals Factors related to women
Perceived consequences of obstetric violence	• In women: Physical and psychological harm Distrust of the healthcare system Perpetuation of the negative childbirth experience • In professionals: Emotional burden and shame Professional demotivation
Strategies and recommendations for prevention and eradication.	Continuing education and training Change in care model Improvement of institutional conditions Promotion of a culture of rights Recognition of the role of obstetric nursing/midwives

### 3.1 General perception and recognition of obstetric violence

The included studies indicate that healthcare personnel generally recognize the existence of obstetric violence, although significant nuances, tensions, and contradictions persist regarding its definition, manifestations, and their own involvement. Most professionals, particularly trainees, acknowledge that obstetric violence occurs within their clinical environments and is perpetrated by colleagues. Notably, obstetric nursing students demonstrate a heightened awareness of this issue, conceptualizing it as a form of psychological and physical aggression intrinsically linked to dehumanizing professional practices and maternal suffering. Recognized manifestations include disrespect, the symbolic and physical violation of the female body, and the exercise of clinical power as a form of abuse.

“A violation of her rights as a woman as a pregnant woman, so she loses her rights to autonomy (11)”.

“Above all, it implies the appropriation of bodies and the natural processes of childbirth by the professionals involved in this process… Any treatment towards the client that exposes her or goes against her principles and wishes (12)”.

This recognition holds particular significance within the context of clinical training, where violence is often internalized as part of the socialization process, even in the absence of explicit institutional mechanisms for critical reflection. When witnessing these practices, observers report feelings of discomfort, helplessness, or frustration due to their perceived inability to intervene. This tension between what is taught as ideal and what is experienced in daily practice creates an ethical conflict that can result in emotional distress, professional demotivation, or even the acceptance of violence as the standard of care.

Despite acknowledging obstetric violence as a broader phenomenon, the studies reveal a marked resistance to self-identification as perpetrators. Healthcare professionals frequently rationalize coercive, invasive, or painful procedures as “medically necessary,” “technically justified,” or performed “for the patient's own good.” This clinical rationalization facilitates a subjective disconnection from the category of violence, thereby obstructing internal problematization and accountability.

“Deep breathing during contractions helped in most cases but some patients get out of control towards the end of labour. We are forced to shout and scold them, not too much… but we do not hit them because it is not allowed. Some patients need to be held down when they push the baby out, we do it for their safety (13)”.

The findings of this review indicate that obstetric violence is deeply embedded in clinical practice, contributing to its pervasive yet often unacknowledged presence among both healthcare professionals and patients. The evidence suggests that a significant number of invasive practices are framed as “delivery strategies” or “forms of assistance,” reflecting an uncritical internalization of biomedical models centered on the control of the pregnant body.

Furthermore, some professionals explicitly deny the existence of obstetric violence or challenge the legitimacy of the term, labeling it as inappropriate, offensive, or excessive from a medical and legal perspective. These positions are frequently held by obstetricians who perceive the medical act as inherently protective, a standpoint that obscures the power imbalances and coercive dynamics that permeate obstetric care.

“I can tell you that your question offends me... How can you think I am violent when I bring a human being into the world? It's very easy to criticize when you're not on call with this pressure on you... (14)”.

A tendency to redefine obstetric violence in functional terms was also identified. Specifically, a favorable clinical outcome, defined by the survival of both mother and infant, often serves as a mitigating factor, effectively absolving an intervention of the label of abuse. This “end justifies the means” logic constitutes one of the most complex symbolic barriers to the full recognition of the problem.

In summary, the findings regarding the general perception of obstetric violence reveal a constant tension between the recognition of the phenomenon as a systemic issue and the rationalization of individual practices. Despite growing awareness among trainees and certain professionals, structural, symbolic, and educational obstacles persist, hindering collective problematization. Obstetric violence is not merely exercised; it is learned, legitimized, and reproduced, often remaining unnamed by those who practice it.

### 3.2 Perceived manifestations and types of obstetric violence

The analyzed studies revealed a broad spectrum of obstetric violence manifestations, perceived and narrated by healthcare personnel across various disciplines and training levels. These forms of violence manifest in diverse ways and are frequently justified or obscured within institutional routines, complicating both their identification and eradication. The following section delineates the primary emergent subcategories.

#### 3.2.1 Physical violence

Direct physical violence is one of the most overt and widely recognized forms of abuse perpetrated by healthcare personnel. The reviewed studies indicate the persistence of punitive practices including hitting, slapping, and limb tying, employed to compel cooperation during childbirth. They also mention the imposition of the lithotomy position, which is often justified by professional convenience rather than clinical necessity, despite the significant discomfort and physiological disadvantage it imposes on the patient.

“Some women don’t put so much effort when they are asked to push because of the pain they feel, so the midwives have to cause them pain sometimes by blocking their nostrils, so they would know how the baby feels as a result of their low effort in pushing (15)”.

Consistent reports indicate that invasive procedures have been performed without informed consent. These procedures include repetitive vaginal examinations by multiple professionals, routine episiotomies, the Kristeller maneuver, indiscriminate use of oxytocin, and unnecessary cesarean sections. Furthermore, several testimonies allude to practices that constitute forms of obstetric sexual intrusion, such as unjustified genital examinations or incisions performed without regard for the patient's dignity or bodily autonomy.

“That they are constantly being examined, that one person comes and touches them, then another comes and touches them, without a single person being in charge of their care from the moment they arrive until the end, but rather everyone examines them, that is obstetric violence (16)”.

#### 3.2.2 Verbal and psychological violence

Verbal and psychological violence are among the most prevalent forms identified, often functioning as a widely accepted norm within clinical settings. Studies describe instances of shouting, scolding, and the use of sarcastic or offensive comments, as well as the tendency to blame women for “failing” to follow instructions, “not pushing properly,” or not immediately bonding with the newborn. In certain cases, patients face the threat of abandonment or the denial of assistance if they do not comply with staff demands. A recurrent phenomenon is the systematic disregard of the woman in labor, manifested through a failure to address her requests, withholding information, or the minimization of her emotional state. The imposition of authority, dehumanizing treatment, and breaches of confidentiality were identified as structural elements pervasive in obstetric services.

“The patient sometimes... screams during childbirth... and they ask her not to scream, that when she did it she didn't cry, she didn't scream, that next year she'll be here again to give birth... it's more like a form of insult, really...today I witnessed this myself (17)”.

“The way the doctor spoke… The impatience because childbirth itself takes a lot! The moment that was supposed to be pleasurable turns out to be painful. It is remarkable! I felt sad for what the patient heard… Those verbal aggressions are very bad! It marked me (18)”.

#### 3.2.3 Neglect and omission of care

Another reported manifestation is negligence, defined as the deliberate or systematic omission of care. This includes ignoring requests for clinical assistance or pain management. Furthermore, studies identified practices such as preventing early skin-to-skin contact, failing to facilitate immediate breastfeeding, denying the presence of a companion during labor, and refusing timely admission to obstetric units. While these omissions are sometimes justified by work overload, they reflect an institutional logic that prioritizes procedures over women's needs.

“Not listening clearly to the user and her needs, sometimes ‘downplaying’ her pain or concerns. Ignoring the pain of the user in labor (19)”.

#### 3.2.4 Stigmatization and discrimination

Several studies highlight discriminatory and stigmatizing practices targeting specific populations, including adolescents, women living with HIV, those with a history of substance abuse, and women who are obese or have sought abortion care. Furthermore, ethnicity and socioeconomic status significantly predispose women to violent or negligent treatment. This systemic bias is particularly evident in cases involving Indigenous women, women of African descent, and those from rural communities. Although these forms of violence often remain subtle or unacknowledged, they are deeply rooted in provider attitudes and directly influence how a woman's suffering is either prioritized or delegitimized during clinical care.

“It is easy to abuse obese women. Personal hygiene is a factor too. They come with skin diseases at times. No one wants to touch them. They have to hear a lot of bad comments lying on the labour table… they do not bathe and we have to clean everything as the baby will be born in the unhygienic passage… wood sellers, coal sellers, Bihari women are very dirty. We do not care if one has shaved or not. Many mothers come after trimming. Looks like they have come straight from the parlour, freshly waxed. They get good care, we like touching them (20)”.

#### 3.2.5 Excessive interventionism and medicalization

Finally, excessive interventionism and the medicalization of childbirth were identified as structural pillars of obstetric violence. Most studies caution against the pathologization of physiological processes and the application of rigid protocols and unnecessary procedures. Notably, some of these interventions are performed for pedagogical purposes without regard for the patient's rights or informed consent. An institutional culture that prioritizes technical proficiency and control over the pregnant body perpetuates these practices, often in the absence of critical reflection by healthcare providers.

“Often, the patient is given medication to dilate the uterus or vagina without her consent or when she does not want analgesia, but it is done anyway, so somehow the human side is lost (16)”.

“While it is true that teaching hospitals are necessary for students to learn, it is unacceptable for everyone who passes by to perform a vaginal examination… I feel that it is not a suitable place for childbirth, and the mother’s dignity is compromised in this system (21)”.

### 3.3 Factors contributing to obstetric violence

Obstetric violence cannot be understood in isolation or reduced to the individual responsibility of practitioners. The analyzed studies reach a consensus that obstetric violence is a structural phenomenon driven by multiple interrelated factors, including institutional conditions, health policies, clinical socialization, professional belief systems, and the intersectional characteristics of the patients. The primary contributing factors are detailed below.

A prominent finding is the systemic pressure on healthcare professionals to guarantee “successful” births, a term primarily defined by the absence of maternal or fetal mortality. This outcome-oriented pressure often precipitates the adoption of interventionist or violent practices as a means to ensure a favorable clinical result. Compounding these pressures are work overload, physical and mental exhaustion, and high stress levels in contexts of understaffing, prolonged shifts, and excessive workloads. Furthermore, precarious institutional conditions including inadequate infrastructure, a shortage of beds, and a lack of essential medical supplies, force professionals into situations of improvisation. This systemic neglect compromises the quality of care and fosters an environment of frustration where violence is often displaced onto the patient.

“So I think the greatest violence is actually not having minimal conditions for the patient to be assisted, it needs to start having a good maternity, graduated and qualified professionals, this will be exchanged in safety (22)”.

The hegemony of the biomedical model over other disciplines, such as nursing and midwifery, as well as over the experiential knowledge of women, establishes rigid hospital hierarchies. These structures foster vertical relationships that stifle shared decision-making and relegate certain professionals to the role of “silent witnesses”, effectively discouraging the questioning of violent practices. Furthermore, the rigid application of standardized protocols, which often fail to account for individual patient needs, promotes routine, mechanical, and depersonalized care. Continuous exposure to childbirth within these inflexible institutional frameworks reinforces a technocratic approach, contributing to the normalization of violence and a profound erosion of empathy within the provider-patient relationship.

“The reality is that you can get into the work with the best intentions, but when you are trained under and within this system, when you are working within this system—well, the issue is the system. It is not always the individual; it is not always the people; it is the system they have to follow. They follow policy, not nature (23)”.

Other structural factors include the pervasive fear of litigation or professional sanctions for medical errors, which fosters a culture of preventive medicalization and excessive control. Physicians often feel compelled to practice defensive medicine, prioritizing procedures that are legally defensible or viewed favorably by peers and judicial systems over those that align with the patient's preferences. Similarly, several studies highlight the systemic lack of professional recognition for obstetric nursing and midwifery. This institutional invisibility undermines their professional autonomy and marginalizes their participation in clinical decision-making, reinforcing the hierarchical and interventionist nature of obstetric care.

“Well [when I have witnessed obstetric violence], I have felt violent, violent and ashamed, the times I have been able to say something, but obviously if it is a senior doctor who has been there for a thousand years and is above you, obviously you're not going to say anything to him, what are you going to say, if he’s not going to change the way he acts either (24)”.

Academic training emerges as a critical factor in the perpetuation of obstetric violence. Multiple studies identify a significant deficit in education regarding the humanization of childbirth, sexual and reproductive rights, and adherence to current national and international regulations, such as Mexican Official Norm 007 (NOM-007) and WHO guidelines. A gap exists between theoretical ethics and clinical reality, creating cognitive and ethical dissonance in students when they encounter environments that contradict classroom teachings. This contradiction often leads to initial feelings of discomfort and frustration; however, without institutional support for critical reflection, it frequently culminates in the long-term normalization of violent practices as students adapt to the prevailing clinical culture.

“I don't have any knowledge about obstetric violence; this discussion was not part of my training. Today they want to change all the existing routines. Many talk about safety, but will it be safe for them (women)? (25)”.

Beyond structural factors, some studies emphasize the role of personal attitudes and professional beliefs in reproducing obstetric violence. Documented evidence reveals a lack of empathy, patience, and sensitivity, as well as moral or personal prejudices toward women, particularly regarding their sexuality, parity, or “behavior” during childbirth. A recurrent theme in these discourses is a reductionist view of the female body, which is frequently perceived as inherently weak, defective, or incapable of physiological birth without intensive medical intervention. This perception justifies aggressive interventions as a means of “helping” or “correcting” women. In this context, violence becomes a functional strategy for achieving a “successful” delivery.

“Look, honey, if you don't want your child to be disabled or something, push because the baby is already here, you can already see its little black face, the baby has to breathe now'. I know that what I did is obstetric violence, but you also have to understand that you're there in the moment, the baby has to come out, she has to push it out, we can't do it for her, and yes, look, it worked because she pushed and pushed until it came out; it's obstetric violence and sometimes we do it without meaning to (16)”.

Finally, certain studies indicate that factors associated with the women themselves significantly influence the quality of care received, as perceived by healthcare personnel. For instance, a perceived lack of knowledge regarding the physiological birthing process and reproductive rights is noted to increase women's vulnerability to abusive practices. Furthermore, women's behavior during childbirth is frequently filtered through a disciplinary lens: those who express fear, scream, or struggle to comply with instructions are labeled as “difficult” or “uncooperative.” This labeling often serves as a catalyst for violent or punitive responses from staff. Such a perspective perpetuates a logic of control, wherein the “ideal patient” is defined by her passive submission to professional authority and the suppression of her own agency.

“Another situation, also related to not staying still during stitches, during suturing, involved a young girl, 16 years old, it was her first child... and she said they had given her anesthesia and everything, but she was afraid, everything was new to her, and she wouldn't stay still, she kept moving her butt, lifting it up, so the professional left, dropped everything and said, ‘Until you stay still, I'm not coming back’ (26)”.

### 3.4 Consequences of obstetric violence

Obstetric violence exerts a profound impact not only on the women who endure it but also on the healthcare professionals who perpetrate, witness, or perpetuate these practices. The reviewed studies identify multifaceted consequences encompassing both physical and emotional spheres, underscoring the severity and complexity of this phenomenon within obstetric care.

#### 3.4.1 Consequences for women

The literature consistently indicates that women subjected to obstetric violence suffer a wide spectrum of physical and psychological repercussions. These range from acute emotional distress to severe clinical conditions, including postpartum depression, post-traumatic stress disorder (PTSD), anxiety, and pervasive feelings of frustration, shame, and helplessness. These effects tend to persist long-term, particularly when the violence is systematic or institutionally denied. Furthermore, some studies document cases where violence during childbirth triggers a rejection of the newborn or profound, irrational feelings of guilt. Such findings highlight the devastating potential of obstetric violence to disrupt the maternal-infant bond during its most critical stages of formation.

“[Obstetric violence causes] depression, stress, panic attacks, low self-esteem, anxiety, loneliness, anger, helplessness... rejection of the newborn, or blaming them for the suffering received (19)”.

The impact of these negative experiences extends far beyond the individual level. Many women disseminate accounts of obstetric violence within their communities, fostering a collective narrative that characterizes childbirth as an inherently painful, dangerous, and humiliating ordeal. This social transmission of trauma shapes the expectations and fears of future expectant mothers, potentially deterring them from seeking timely professional care.

#### 3.4.2 Consequences for health professionals

The effects of obstetric violence extend to those who perpetrate or witness it, particularly when there is a discrepancy between the ethical principles taught in professional training and the actual practices of institutions. Several studies report that professionals, particularly nursing and midwifery students and staff, experience heavy emotional burdens and feelings of shame, helplessness, or guilt when they witness or participate in unjustified violent practices but do not feel in a position to question them.

“There was one occasion when I was forced to perform an episiotomy, even though I believed it was unnecessary. I felt terrible (27)”.

The disconnect between the competencies acquired during training and the reality of hospital environments can precipitate a profound internal conflict. This often manifests as professional demotivation, cynicism, or abandonment of the vocation. Some even develop symptoms of secondary traumatic stress, particularly when they have witnessed intense and repeated incidents within a culture of institutional silence. Furthermore, some physicians perceive themselves as victims of violence within the medical landscape. This perception stems from two primary sources: institutional violence, characterized by precarious working conditions, systemic resource scarcity, and excessive workloads; and interpersonal violence from patients and their families, manifested through threats, hostility, or overt contempt regarding clinical decisions.

“To work without having enough hospital beds available, overburdened nursing staff, citizens dissatisfied with the public health system, and a culture in which “the doctor is to be blamed for everything (28)”.

This complex dynamic suggests that obstetric violence occurs within a broader ecosystem of precariousness that affects all actors involved. The systematic reproduction of a dehumanized model of care progressively erodes their professional subjectivity and their fundamental capacity for compassionate care, creating a cycle of institutional violence that is difficult to break without structural intervention.

### 3.5 Strategies and recommendations to combat obstetric violence

Throughout the reviewed studies, multiple proposals emerge aimed at transforming obstetric environments and preventing the reproduction of violent practices. These strategies, developed by practitioners and through institutional and pedagogical analyses, emphasize an urgent need for cultural, structural, and epistemological shifts to guarantee respectful care centered on women's rights.

A predominant theme in the recommendations is the necessity of a comprehensive overhaul of training processes, encompassing both undergraduate education and continuous professional development. There is a strong emphasis on promoting curricula grounded in evidence-based medicine and a humanized approach. This includes the integration of explicit content on human rights, sexual and reproductive health, bodily autonomy, and gender perspectives

“I think that sometimes it is due to a lack of information at university, to the way it was taught, because doctors often do what they were taught; that is how they learned in their academic life. It is about changing the basis of teaching, and this is what is changing now: gynecology and obstetrics courses are delving into the area of care, of humanized childbirth, and end up teaching how to treat and approach, because sometimes it can be done without the notion that this is violence, because they were taught to do it, and this is a multi-professional problem, also in nursing and pediatrics (22)”.

Similarly, the studies propose a reformulation of curricular matrices to ensure that future professionals are not only technically proficient but also ethically empowered to practice with empathy and respect. Fostering communication skills, active listening, and interdisciplinary teamwork from the onset of training is considered indispensable for transforming the vertical power dynamics inherent in obstetric care. Consequently, a critical priority is the transition from a technocratic, interventionist model toward a woman-centered model of care. This paradigm shift necessitates a revaluation of the physiology of childbirth, the formal recognition of the patient's autonomy, and the active promotion of her participation in clinical decision-making.

“I think it scares us because we come from a paternalistic and sexist healthcare system, where we are used to making decisions for others... for us, it is much easier to believe that what we are doing is what happens most often, and we come from the idea that we have the knowledge and the other person does not, so we are in this position of power: I know and you don't. And that's how it is, I think, it scares us to know that we don't know as much as we think we do, and that, in the end, we are affecting a person who has the right to decide what is best for them and not for us (29)”.

Some of the interviewed professionals recommend eliminating practices that lack scientific evidence or are clearly harmful, such as routine episiotomies, the Kristeller maneuver, and denying support. They also recommend creating institutional guidelines that support these changes.

Furthermore, there is a consensus that no transformation will be sustainable without substantive improvements in material and institutional conditions. Essential recommendations include ensuring adequate infrastructure, the consistent availability of supplies, and a sufficient ratio of qualified staff to patients. Creating physical environments that guarantee privacy and dignity during labor is also paramount. Additionally, proposals include optimizing shift management to reduce work overload and establishing effective supervision mechanism**s** designed for continuous quality improvement and the timely correction of malpractice.

“Preparedness of the entire team of doctors, nurses, technical staff to manage work according to the new guidelines on labor, as well as a physical structure of the maternity ward adapted to humanized childbirth (30)”.

Real transformation involves actively promoting a culture of rights both within and outside the hospital setting. Studies suggest raising awareness among institutions, women, and society regarding obstetric violence and its severity. Additionally, educational campaigns and training spaces should be developed to allow for collective reflection on this phenomenon.

“Improvement of prenatal care, better educate of the citizens, improve hospital structure and training of the entire staff (30)”.

The studies emphasize creating accessible, secure, and permanent reporting channels accompanied by effective follow-up measures. They also advocate for empowering women by educating them about their rights and implementing humanized childbirth policies. These actions prevent violence and promote the development of more symmetrical relationships between healthcare personnel and users.

Finally, underscore the significance of acknowledging and bolstering the role of obstetric nurses and midwives, who frequently cultivate closer, longer-lasting, and more empathetic relationships with women during childbirth. They play a fundamental role in promoting humanized care and defending women's rights, especially in contexts where the biomedical model hinders this vision.

“I think the work is important, but it is not recognized! It is a struggle for nurses to be able to ‘give birth'! It is not recognized by the system, the medical profession, the institution, the population...  (18)”.

### 3.6 Perception of obstetric violence by discipline and level of experience

When analyzing the articles, notable differences can be observed in the perception and experience of obstetric violence across various disciplines (nursing, midwifery, and gynecology-obstetrics) and different levels of experience (professionals vs. students). While the studies agree on the existence of obstetric violence, perspectives vary according to role and training, as summarized in Table 4.

**Table 4:** Summary of disciplinary and experience-based differences in how health personnel perceive and experience obstetric violence.

Characteristic	Obstetrician-Gynecologists (Physicians)	Nursing (General/Technical)	Obstetric Nurses/Midwives (Specialized)	Students (All Disciplines)
Perception of VO	Lack of knowledge/rejection of the term; Workload/pressure as justification.	They are witnesses and participants; Justification due to precarious conditions.	Greater knowledge of the concept; Emphasis on humanization.	Greater sensitivity and initial recognition; Exposure to entrenched practices.
Role in the System	Highest technical-scientific authority; Center of the biomedical model.	Subordinate to doctors; intermediaries in care.	Promoters of humanized childbirth; Autonomy limited by hierarchy.	Critical observers of current practices; Potential agents of change.
Causal Factors	Pressure for results; Medical justification; Personal convenience.	Patient overload; Lack of resources; Hierarchies.	Biomedicine; Pathologization of childbirth; Hospital hierarchies.	Superficial academic training; Entrenched biomedical model.
Type of Violence	Interventionism; Gynecological position; Lack of information.	Verbal; Negligence; Discrimination.	Physical; Verbal; Psychological; Restriction.	All forms, from subtle to explicit.
Attitude toward Change	Resistance to dialogue; adherence to ancient teachings.	Normalization; Adaptation to the system.	Advocacy for change; Emphasis on scientific evidence; Dialogue.	Identification of deficiencies; Desire to transform care.
Personal Impact	Feel guilty or victimized by the term.	Stress; Burnout; Feeling of complicity.	Feelings of aggression, shame, complicity; Burnout.	Suffering; Demotivation; Ethical dilemmas.

Gynecologists and obstetricians have a specific perception of obstetric violence. Many are unaware of or reject the term, considering it offensive and irrelevant to their practice. They justify their actions as necessary for maternal and fetal safety. Conversations often focus on workload, institutional pressures, and precarious working conditions, which are identified as factors that influence their performance and contribute to professional exhaustion. Routine interventions such as episiotomies, forceps deliveries, and cesarean sections without consent, are often justified as necessary for safety or convenience within a protocolized model. Additionally, some doctors perceive themselves as victims of either the hospital system or confrontational patients, and their position of maximum hierarchical authority contributes to asymmetrical relationships with women and other professionals.

A dual perception prevails among general and technical nursing staff: they frequently witness obstetric violence perpetrated mainly by doctors, yet they also acknowledge reproducing it, sometimes unconsciously, and normalizing it as part of hospital dynamics. Adverse working conditions, such as patient overload, staff and material shortages, and long shifts, are cited as the main factors that make providing humane care difficult. Verbal abuse and neglect are common occurrences, and hierarchical subordination to medical staff often forces them to be silent witnesses, which reinforces a form of symbolic violence within the hospital structure.

Obstetric nurses and midwives show a clearer awareness of obstetric violence and its link to the need to humanize childbirth. They interpret obstetric violence as a violation of the bioethical principles of professional practice. They explicitly identify violent procedures, including the Kristeller maneuver, routine episiotomies, the indiscriminate use of oxytocin, the restriction of movement or position, and the lack of information and consent. Some acknowledge participating directly in these practices or enabling them as “accomplices” due to training that normalizes such practices. This generates feelings of shame and ethical discomfort. These professionals tend to question the biomedical model's interventionist approach; however, they also note that medical hierarchies limit their autonomy and decision-making capacity. Finally, they propose strategies to improve the situation, including continuous training, strengthening the bond with patients, engaging in professional self-criticism, and actively promoting humane treatment (31).

When analyzing perceptions according to the level of experience, a notable change in sensitivity to obstetric violence emerges. Established professionals tend to normalize or justify certain practices within the institutional framework, while students are more willing to recognize obstetric violence as an ethical and social problem.

Students are more explicitly recognizing obstetric violence, which they associate with disrespect, suffering, and the violation of rights. During their internships and residencies, students are exposed to entrenched, non-evidence-based protocols that cause discomfort and feelings of involuntary complicity (32). They perceive initial training as insufficient for preventing and addressing obstetric violence because it tends to reproduce a technocratic model that pays little attention to humanizing childbirth. This situation raises ethical dilemmas regarding intervening in the face of witnessed violence, a dilemma exacerbated by the fear of reprisals and hospital hierarchy. However, students have the potential to bring about significant change. Their critical view and lesser attachment to traditional practices open the door to rethinking teaching programs and promoting more respectful, woman-centered obstetric care.

Among more experienced professionals, there is a tendency to normalize and justify obstetric violence. They perceive it as a “natural” consequence of the care process or as an inevitable result of the limitations of the health system, which are marked by staff shortages, excessive workload, and poor infrastructure. They resist change based on their confidence in their training and clinical experience, even when it conflicts with current scientific evidence. Some professionals, especially nurses and midwives, report having witnessed or experienced violence in their workplace, primarily verbal or psychological abuse by supervisors, reflecting the perpetuation of violent dynamics within the healthcare system.

No studies documenting general practitioners' perceptions of obstetric violence were found in the reviewed literature. This absence is significant because these professionals are often the first point of contact for pregnant women and play a key role in detecting, preventing, and providing initial care for situations of violence.

## 4 Discussion

Obstetric violence is increasingly framed within international literature as “obstetric mistreatment” or “disrespect and abuse.” The findings of this qualitative systematic review must be interpreted in dialogue with a burgeoning body of empirical intervention research, which posits that this phenomenon is predominantly systemic and modifiable rather than merely the aggregate of isolated individual behaviors. Recent evidence, including a 2024 scoping review, suggests that structured interventions at the facility level can effectively recalibrate professional attitudes and mitigate harmful practices by reshaping organizational norms, communication patterns, and care models (33). Furthermore, studies utilizing validated perception-based instruments, such as the PercOV-S (34), alongside pre–post intervention designs, demonstrate that institutionally driven educational and accreditation initiatives can significantly shift healthcare workers' perceptions toward mistreatment.

Evidence from institutional accreditation frameworks, such as the Mother–Baby Friendly Facility model evaluated by Tercan et al. (35), further indicates that when medico-legal concerns, clinical safety, and respectful care are addressed concurrently through standardized institutional protocols, the perceptions of both physicians and midwives shift in meaningful ways. This suggests that shifting from an individual-blame perspective to a systems-thinking approach is crucial for sustainable reform.

Taken together, these findings reinforce the necessity of conceptualizing obstetric violence and mistreatment as phenomena deeply embedded within organizational culture, professional socialization, and structural constraints, rather than as products of individual intent alone. This paradigm shift allows us to move beyond attributing the dismissive narratives identified in the reviewed articles solely to personal resistance or a lack of empathy. Instead, these attitudes can be understood as responses to systemic stressors, including defensive medicine, increasing medico-legal pressure, the burden of responsibility in high-risk clinical scenarios, and the normalization of emergency-oriented decision-making inherent in traditional obstetric training.

This qualitative systematic review provides valuable insights into healthcare personnel's perceptions of obstetric violence. Among its primary strengths is the hermeneutic approach applied to the analysis, which facilitates an in-depth recovery of the meanings and lived experiences reported across diverse contexts. Furthermore, the predominance of research from Latin America offers a situated overview, highlighting the sociocultural particularities of the region and the specific ways they shape obstetric care. The synthesis achieved through rigorous qualitative methodologies enables the identification of recurrent patterns and emerging questions that significantly enrich our understanding of this phenomenon.

However, it is important to note some limitations. The absence of studies that include the voices of certain professionals, such as general practitioners, limits the breadth of the analysis and leaves areas of literature largely unexplored. Similarly, the predominance of studies from Latin America, particularly from countries like Brazil, Mexico, and Chile, significantly influences the findings and may limit their transferability to other healthcare systems due to specific regional medical cultures and legal frameworks. The Latin American obstetric model is frequently characterized as a “technocratic” or highly interventionist system where childbirth is often viewed as a pathological event requiring excessive medicalization (24). For instance, Brazil reports C-section rates as high as 85% in private health services and high frequencies of routine interventions like episiotomies and the Kristeller maneuver (17). This specific context may not directly translate to healthcare systems with different paradigms, such as those with stronger independent midwifery models or lower baseline intervention rates found in some European settings (20, 21).

Furthermore, the structural and economic drivers of obstetric violence reported in the included studies vary by region, affecting generalizability. While Latin American studies often focus on the abuse of medical authority and institutional overcrowding, studies from Ghana and South Africa highlight extreme structural forms of violence, such as detaining women in facilities for unpaid bills or the extortion of “egg money” by staff (15). Additionally, social determinants of respectfulness, such as caste-based discrimination in India, introduce unique intersectional dynamics that may not be fully captured in Latin American qualitative research focused primarily on gender and medical hierarchy (20).

The concentration of studies from Latin America may be related to the terms used in the search strategy. The concept of *obstetric violence* is more widely recognized in this region, both academically and legally, as well as within activist circles. This makes it easier to identify works that explicitly use the term. In contrast, Global North still prefers expressions such as *substandard care, disrespectful and abusive care*, or *mistreatment during childbirth* (36). While these terms address similar phenomena, they have been adopted partly to circumvent the political and structural connotations associated with the concept of violence.

This terminological divergence reflects semantic, epistemological, and political differences related to acknowledging the structural, institutional, and gendered dimensions inherent in labeling these practices as violent. Thus, the limited presence of studies using the term “obstetric violence” outside of Latin America does not necessarily indicate the absence of the phenomenon. Rather, it suggests the persistence of analytical frameworks that soften or shift the debate toward categories that are considered more acceptable within hegemonic health systems.

## 5 Conclusion

This qualitative systematic review examined how healthcare personnel perceive and experience obstetric violence. The findings reveal that these perceptions are shaped by hierarchical power structures, institutional values, and educational backgrounds. Although there is growing recognition of the phenomenon, the findings show that mechanisms of normalization and justification persist, rendering obstetric violence invisible and perpetuating it in everyday clinical practice.

The analysis highlights significant differences in sensitivity and critical awareness between disciplines and levels of experience. Students and midwives demonstrate greater sensitivity and critical awareness, while experienced professionals, especially physicians, tend to justify interventionist practices and normalize violence as part of the hospital routine or an inevitable consequence of the health system's shortcomings. This approach allows us to understand healthcare personnel as social actors who are immersed in a system that reproduces dynamics of domination and structural violence. This system generates ethical dilemmas and emotional distress, particularly among trainees.

Based on this evidence, there is a clear need for comprehensive interventions that include the following:

Curricular reforms and continuous training focused on human rights, gender perspectives, and the humanization of childbirth.
Improvements in institutional conditions to ensure adequate infrastructure, sufficient staffing, and safe spaces for emotional and physical support.
Clear public policies and regulations that promote shared responsibility and highlight obstetric violence as a public health and human rights issue.

This review emphasizes the necessity of transforming obstetric care by acknowledging the voices of healthcare personnel, understanding the structural conditions that influence their practices, and promoting educational, organizational, and sociocultural shifts. Such changes are essential to eradicate obstetric violence and ensure that all women receive respectful, safe, and dignified care.

## Data Availability

The original contributions presented in the study are included in the article/Supplementary Material, further inquiries can be directed to the corresponding author.
